# Supplementary material for: Functional and evolutionary significance of unknown genes from uncultivated taxa
Source: Nature. 2023 Dec 18;626(7998):377–84. doi: 10.1038/s41586-023-06955-z (PMC10849945; doi:10.1038/s41586-023-06955-z)
Supplement: Supplementary file 1 — Reporting Summary [file 41586_2023_6955_MOESM1_ESM.pdf]

nature portfolio

Corresponding author(s): Jaime Huerta Cepas

Last updated by author(s): 26 Nov, 2023

Reporting Summary

Nature Portfolio wishes to improve the reproducibility of the work that we publish. This form provides structure for consistency and transparency in reporting. For further information on Nature Portfolio policies, see our Editorial Policies and the Editorial Policy Checklist.

Statistics

For all statistical analyses, confirm that the following items are present in the figure legend, table legend, main text, or Methods section.

n/a	Confirmed	
☐	☒	The exact sample size (*n*) for each experimental group/condition, given as a discrete number and unit of measurement
☐	☒	A statement on whether measurements were taken from distinct samples or whether the same sample was measured repeatedly
☐	☒	The statistical test(s) used AND whether they are one- or two-sided<br>*Only common tests should be described solely by name; describe more complex techniques in the Methods section.*
☒	☐	A description of all covariates tested
☐	☒	A description of any assumptions or corrections, such as tests of normality and adjustment for multiple comparisons
☐	☒	A full description of the statistical parameters including central tendency (e.g. means) or other basic estimates (e.g. regression coefficient) AND variation (e.g. standard deviation) or associated estimates of uncertainty (e.g. confidence intervals)
☐	☒	For null hypothesis testing, the test statistic (e.g. *F*, *t*, *r*) with confidence intervals, effect sizes, degrees of freedom and *P* value noted<br>*Give P values as exact values whenever suitable.*
☒	☐	For Bayesian analysis, information on the choice of priors and Markov chain Monte Carlo settings
☒	☐	For hierarchical and complex designs, identification of the appropriate level for tests and full reporting of outcomes
☒	☐	Estimates of effect sizes (e.g. Cohen's *d*, Pearson's *r*), indicating how they were calculated

Our web collection on statistics for biologists contains articles on many of the points above.

Software and code

Policy information about availability of computer code

Data collection	No specific software was used to collect data. Original datasets were manually downloaded from their public sources.
Data analysis	MMseqs2, version, https://github.com/soedinglab/MMseqs2<br>diamond, version v0.9.32.133, https://github.com/bbuchfink/diamond<br>HMMER, version 3.2, http://hmmer.org/<br>clustalO, version 1.2.4: http://www.clustal.org/omega/<br>FastTree, version 2.1, : http://www.microbesonline.org/fasttree/<br>ETE3, version 3.1.2, : http://etetoolkit.org/download/<br>HyPhy, version 2.5.14, : https://www.hyphy.org/<br>RNAcode, version 0.3: https://github.com/ViennaRNA/RNAcode<br>mongoDB, version 3, : https://www.mongodb.com/<br>GTDB-Tk, version v1.6.0,: https://github.com/Ecogenomics/GTDBTk<br>Macrel, version 0.6.1, https://github.com/BigDataBiology/macrel<br>Seeker, https://github.com/gussow/seeker<br>Plasflow, version 1.1, https://github.com/smaegol/PlasFlow<br>SignalP, version 5.0, https://services.healthtech.dtu.dk/services/SignalP-5.0/<br>TMHMM version 2.0. https://services.healthtech.dtu.dk/services/TMHMM-2.0/<br>Alistat, http://www.csb.yale.edu/userguides/seq/hmmer/docs/node27.html

For manuscripts utilizing custom algorithms or software that are central to the research but not yet described in published literature, software must be made available to editors and reviewers. We strongly encourage code deposition in a community repository (e.g. GitHub). See the Nature Portfolio guidelines for submitting code & software for further information.

Data

Policy information about availability of data

All manuscripts must include a data availability statement. This statement should provide the following information, where applicable:

- Accession codes, unique identifiers, or web links for publicly available datasets
- A description of any restrictions on data availability
- For clinical datasets or third party data, please ensure that the statement adheres to our policy

All genomic data used in this study were downloaded from public sources as follows:
UGHH MAGs from https://www.ebi.ac.uk/ena/browser/view/PRJEB33885, Ocean MAGs and SAGs from https://www.ebi.ac.uk/ena/browser/view/PRJEB45951 and https://microbiomics.io/ocean/, GMGC MAGs from https://gmgc.embl.de, GEM MAGs from https://genome.jgi.doe.gov/GEMs, and GTDB reference genomes and MAGs from https://data.gtdb.ecogenomic.org/releases/release95/95.0. All the derived results from this study, including FESNov gene families fasta files, phylogenetic trees and alignments, per FESNov gene family statistics and evolutionary information, mobile element detections, taxonomic annotations, functional prediction summaries and protein structure predictions are available at https://zenodo.org/doi/10.5281/zenodo.10219528. All genomic data used in this study were downloaded from public sources as follows:
UGHH MAGs from https://www.ebi.ac.uk/ena/browser/view/PRJEB33885, Ocean MAGs and SAGs from https://www.ebi.ac.uk/ena/browser/view/PRJEB45951 and https://microbiomics.io/ocean/, GMGC MAGs from https://gmgc.embl.de, GEM MAGs from https://genome.jgi.doe.gov/GEMs, and GTDB reference genomes and MAGs from https://data.gtdb.ecogenomic.org/releases/release95/95.0. All the derived results from this study, including FESNov gene families fasta files, phylogenetic trees and alignments, per FESNov gene family statistics and evolutionary information, mobile element detections, taxonomic annotations, functional prediction summaries and protein structure predictions are available at https://zenodo.org/doi/10.5281/zenodo.10219528. Computer generated structural models are also available at https://modelarchive.org/doi/10.5452/ma-fesnov. In addition, large intermediate files from some analyses are provided at https://novelfams.cgmlab.org/downloads/, including: all the standardized genomes, MAGs and SAGs downloaded from public sources, consolidated FASTA files with predicted genes and proteins, functional annotations of all proteins by eggNOG-mapper v2.1 and raw clustering results.
In addition, large intermediate files from some analyses are provided at https://novelfams.cgmlab.org/downloads/, including: all the standardized genomes, MAGs and SAGs downloaded from public sources, consolidated FASTA files with predicted genes and proteins, functional annotations of all proteins by eggNOG-mapper v2.1 and raw clustering results.

Research involving human participants, their data, or biological material

Policy information about studies with human participants or human data. See also policy information about sex, gender (identity/presentation), and sexual orientation and race, ethnicity and racism.

Reporting on sex and gender	N/A
Reporting on race, ethnicity, or other socially relevant groupings	N/A
Population characteristics	N/A
Recruitment	N/A
Ethics oversight	N/A

Note that full information on the approval of the study protocol must also be provided in the manuscript.

Field-specific reporting

Please select the one below that is the best fit for your research. If you are not sure, read the appropriate sections before making your selection.

☐ Life sciences ☐ Behavioural & social sciences ☒ Ecological, evolutionary & environmental sciences

For a reference copy of the document with all sections, see nature.com/documents/nr-reporting-summary-flat.pdf

Ecological, evolutionary & environmental sciences study design

All studies must disclose on these points even when the disclosure is negative.

Study description	A metagenomics study on the discovery and characterization of novel microbial gene families with significant functional, ecological and evolutionary vcalue.
Research sample	A collection of over 140,000 MAGS collected from various public studies.
Sampling strategy	N/A
Data collection	149,842 medium and high-quality metagenome assembled genomes (MAGs) and single-amplified genomes (SAGs), alongside 19,642 reference genomes from isolated and fully sequenced species. This set includes over 400 million gene predictions and was assembled

	by unifying five distinct data sources spanning 82 habitats: two MAG collection spanning thousands of samples from diverse origins (GEM and GMGC), a comprehensive human gut catalog (UHGG), a global ocean catalog (OMD), and the GTDB r95 reference database (Table S1).
Timing and spatial scale	N/A
Data exclusions	Low quality MAGs and spurious sequences removed.
Reproducibility	All available data and scripts to reproduce our analyses are provided as supplementary material and as an online repository.
Randomization	N/A
Blinding	N/A

Did the study involve field work? ☐ Yes ☒ No

Reporting for specific materials, systems and methods

We require information from authors about some types of materials, experimental systems and methods used in many studies. Here, indicate whether each material, system or method listed is relevant to your study. If you are not sure if a list item applies to your research, read the appropriate section before selecting a response.

Materials & experimental systems

n/a	Involved in the study
☒	☐ Antibodies
☒	☐ Eukaryotic cell lines
☒	☐ Palaeontology and archaeology
☒	☐ Animals and other organisms
☒	☐ Clinical data
☒	☐ Dual use research of concern
☒	☐ Plants

Methods

n/a	Involved in the study
☒	☐ ChIP-seq
☒	☐ Flow cytometry
☒	☐ MRI-based neuroimaging

Plants

Seed stocks	N/A
Novel plant genotypes	N/A
Authentication	N/A
